# Pseudo-Wellens syndrome, acute pancreatitis, and an anomalous coronary artery: a case report

**DOI:** 10.1186/s13256-019-2315-1

**Published:** 2019-12-30

**Authors:** V. S. Effoe, W. O’Neal, R. Santos, L. Rubinsztain, A. M. Zafari

**Affiliations:** 1 Atlanta Veterans Health Care System, Decatur, GA USA; 2 Department of Medicine, Morehouse School of Medicine, Atlanta, GA USA; 3 Division of Cardiology, Department of Medicine, Emory University School of Medicine, Atlanta, GA USA; 4 Department of Radiology, Emory University School of Medicine, Atlanta, GA USA

**Keywords:** Chest pain, Pancreatitis, ECG, Wellens syndrome, Myocardial infarction, Anomalous origin of coronary artery

## Abstract

**Background:**

Chest pain associated with transient electrocardiogram changes mimicking an acute myocardial infarction have been described in acute pancreatitis. These ischemic electrocardiogram changes can present a diagnostic dilemma, especially when patients present with concurrent angina pectoris and epigastric pain warranting noninvasive or invasive imaging studies.

**Case presentation:**

A 45-year-old African-American man with a history of alcohol use disorder presented to the emergency department of our institution with 36 hours of concurrent epigastric pain and left-sided chest pain radiating to his left arm and associated with nausea and dyspnea. On physical examination, he was afebrile; his blood pressure was elevated; and he had epigastric tenderness. His laboratory test results were significant for hypokalemia, normal troponin, and elevated serum lipase and amylase levels. Serial electrocardiograms for persistent chest pain showed ST-segment elevations with dynamic T-wave changes in the right precordial electrocardiogram leads, consistent with Wellens syndrome. He was immediately taken to the cardiac catheterization laboratory, where selective coronary angiography showed normal coronary arteries with an anomalous origin of the right coronary artery from the opposite sinus. Given his elevated lipase and amylase levels, the patient was treated for acute alcohol-induced pancreatitis with intravenous fluids and pain control. His chest pain and ischemic electrocardiogram changes resolved within 24 hours of admission, and coronary computed tomography angiography showed an interarterial course of the right coronary artery without high-risk features.

**Conclusions:**

Clinicians may consider deferring immediate cardiac catheterization and attribute electrocardiogram changes to acute pancreatitis in patients presenting with angina pectoris and acute pancreatitis if confirmed by normal cardiac enzymes and elevated levels of lipase and amylase. However, when clinical signs and electrocardiogram findings are highly suggestive of myocardial ischemia/injury, immediate noninvasive coronary computed tomography angiography may be the best approach to make an early diagnosis.

## Background

Gastrointestinal disturbances causing chest pain have been observed since the early 20th century [1]. The first report of acute pancreatitis and characteristic electrocardiogram (ECG) changes simulating myocardial infarction was published in 1954 [2]. These ischemic ECG changes can present a diagnostic dilemma, especially when patients present with concurrent angina pectoris.

## Case presentation

A 45-year-old African-American man presented to the emergency department (ED) of our institution with over 36 hours of epigastric pain and left-sided chest pain. The patient described his chest pain as a pressure-like sensation, rated 9/10, radiating to his left arm and associated with nausea and dyspnea, but no vomiting or diaphoresis. He denied palpitations, presyncopal symptoms, orthopnea, paroxysmal nocturnal dyspnea, or lower leg swelling. His pain had worsened over the 24 hours preceding his ED visit. His past medical history was significant for alcohol and tobacco use disorders. He had no personal history of hypertension, hyperlipidemia, or diabetes. He was taking no medications at home, and he had no drug allergies. His mother had diabetes, but he had no family history of early coronary heart disease. He lived with his wife and adult children. Of note, the patient was a current drinker consuming at least 1 pint of liquor daily and a current smoker with a 20-pack-year history. He had been treated for gastritis at another health facility in the past year, where he had presented with abdominal pain.

On physical examination in the ED, the patient was in mild distress. His blood pressure (BP) was 156/101 mmHg; his pulse was 75 beats per minute; his respiratory rate 20 breaths per minute; his temperature 36.8 °C; and his body mass index 25.5 kg/m^2^. He appeared anxious and had epigastric tenderness, but he did not have jugular venous distention, carotid bruits, or cardiac murmurs. The rest of his physical examination was unremarkable. Table 1 displays his laboratory test results upon presentation and 12 hours afterward.

**Table 1:** Laboratory test results upon arrival in the emergency department and 12 hours after arrival

Parameter	Reference range	Upon arrival in ED	12 hours after arrival
Troponin I (ng/ml)	0.00–0.03	< 0.03	
B-type natriuretic peptide (pg/ml)	0–99	59	
Lipase (U/L)	13–51	246	346
Amylase (U/L)	28–100	475	
Ethanol (mg/dl)	0–10	< 5	
White blood cells (10^3^/mm^3^)	4–11	7.1	6.0
Hemoglobin (g/dl)	13.7–17.5	14.0	12.4
Hematocrit (%)	40.1–51	42.7	36.7
Platelets (10^3^/mm^3^)	150–400	167	128
Sodium (mmol/L)	136–145	136	138
Potassium (mmol/L)	3.6–5.1	3.1	3.5
Magnesium (mg/dl)	1.6–2.6	1.7	2.0
Creatinine (mg/dl)	0.5–1.2	0.9	0.7
Urea nitrogen (mg/dl)	8–23	5	3
Glucose (mg/dl)	70–110	108	83

*ED* emergency department

**Fig. 1:**
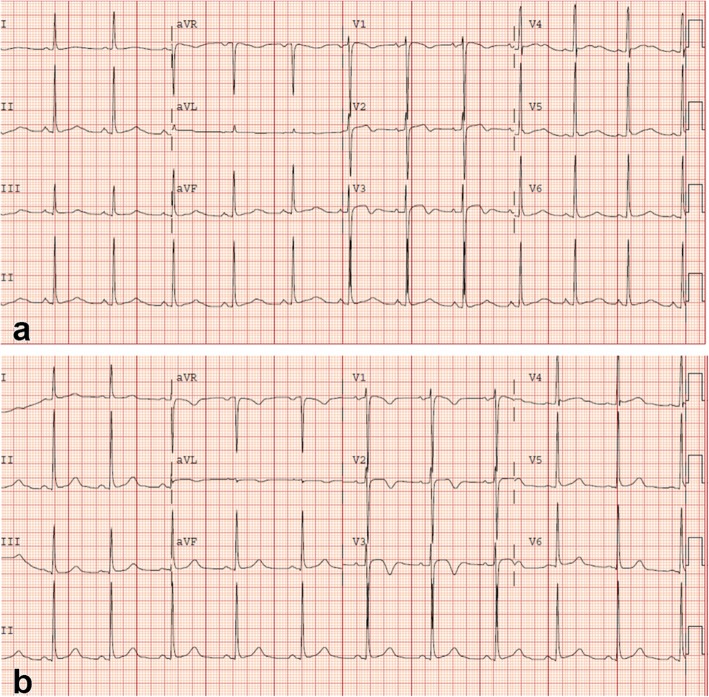
Twelve-lead electrocardiogram obtained upon arrival in the emergency department showing sinus rhythm and 1-mm ST elevations with biphasic T-waves in right precordial leads V_1_–V_3_ (**a**). Twelve-lead electrocardiogram obtained 40 minutes after emergency department arrival showing sinus rhythm and persistent 1-mm ST elevations with pronounced symmetric T-wave inversions in V_1_–V_3_ (**b**)

**Fig. 2:**
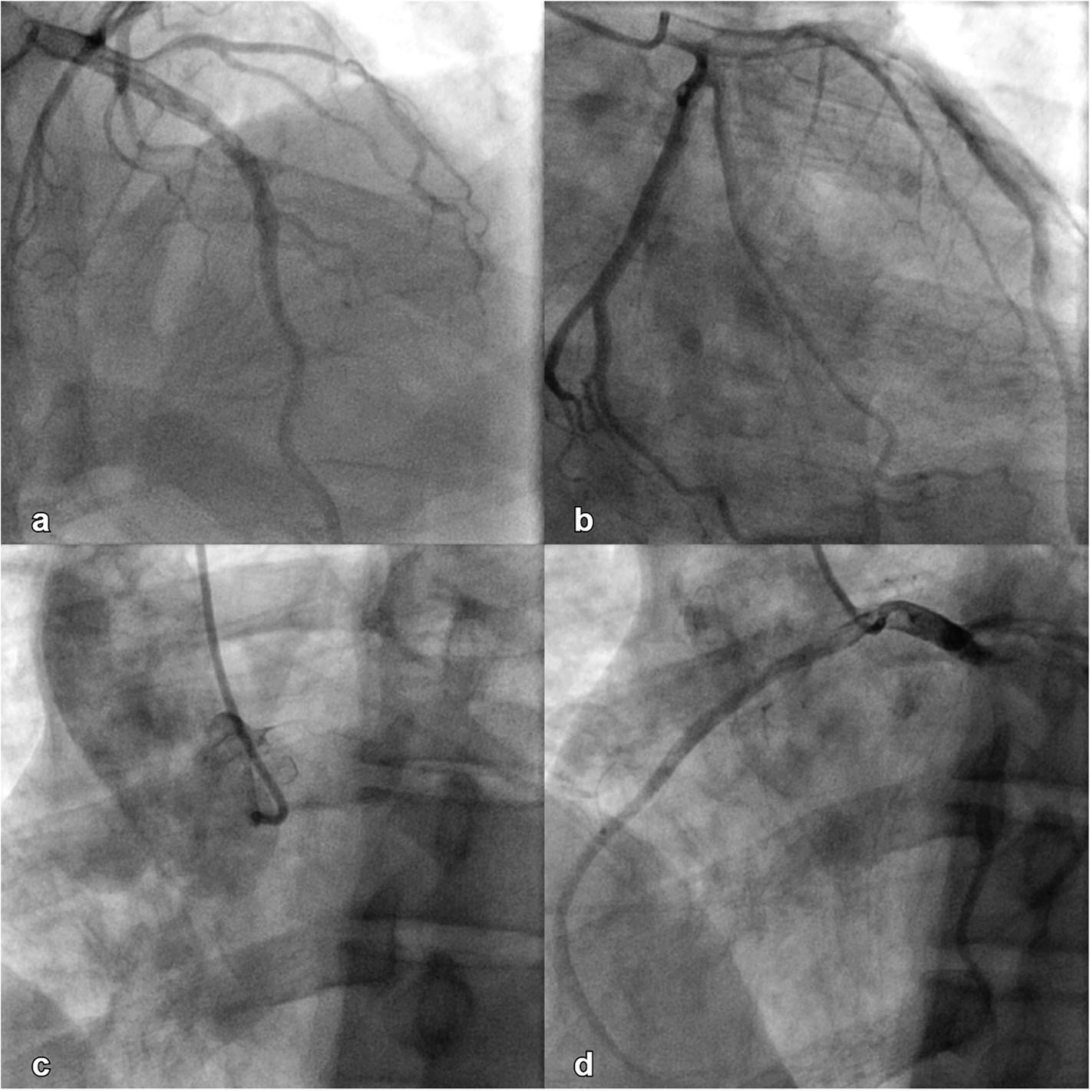
Angiographically normal left anterior descending coronary artery, left anterior oblique projection, cranial angulation (**a**). Angiographically normal left circumflex artery, right anterior oblique projection (**b**). Right coronary cusp injection, left anterior oblique projection (**c**). Angiographically normal right coronary artery originating from the left coronary cusp, left anterior oblique projection (**d**)

An initial ECG performed upon arrival at the ED showed 1-mm ST-segment elevations, T-wave inversions, and biphasic T-waves in his right precordial leads (Fig. 1a). He had persistent chest pain, and a repeat ECG 40 minutes after ED arrival showed persistent ST-segment elevations with dynamic T-wave changes, notably deep symmetric inversions in V_1_–V_3_ (Fig. 1b). Bedside transthoracic echocardiography showed normal left ventricular systolic function and no left ventricular hypertrophy or regional wall motion abnormalities. However, due to persistent chest pain and dynamic ECG changes concerning for critical stenosis high in the left anterior descending (LAD) coronary artery, consistent with Wellens syndrome [3], the patient underwent immediate invasive coronary angiography. Wellens syndrome is characterized by a typical pattern of the ST-T segment in leads V_2_ and V_3_, consisting of an isoelectric or minimally elevated (1-mm) takeoff of the ST segment from the QRS complex, a concave or straight ST segment passing into a negative T wave at an angle of 60 to 90 degrees, a symmetrically inverted T wave, and the absence of pathologic Q waves, in patients presenting with or without angina pectoris and minimal or no elevation of cardiac enzymes. Selective coronary angiography of our patient revealed angiographically normal coronary arteries but an anomalous origin of the dominant right coronary artery (RCA) from the opposite sinus (R-ACAOS) (Fig. 2a–d).

**Fig. 3:**
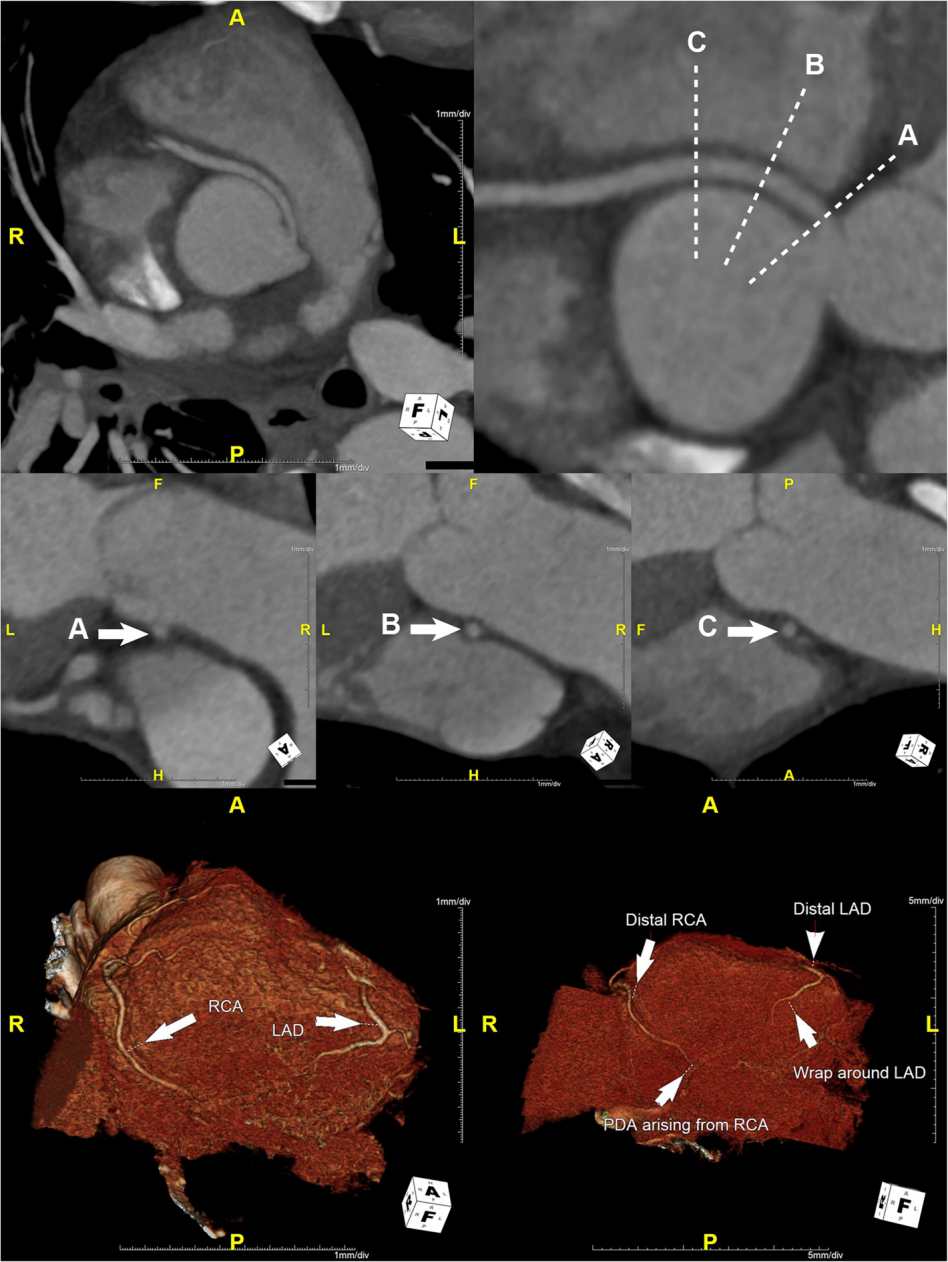
Maximum intensity projection curved planar reformat (CPR) image (top row left) and CPR (top row right) demonstrate an anomalous right coronary artery (RCA) originating from the left coronary sinus with an interarterial course and without evidence of an intramural course. There is no significant stenosis of the ostium of the anomalous RCA. Short-axis CPR images through the proximal anomalous RCA immediately distal to the ostium (middle row A), 1 cm distal to the ostium (middle row B), and 2 cm distal to the ostium (middle row C) demonstrate a patent proximal RCA without evidence of luminal narrowing. 3D volumetric images (bottom row) demonstrate patent distal RCA and left anterior descending (LAD) artery with a small posterior descending artery arising from the distal RCA and with a wrap around distal LAD

He was started on intravenous fluids and pain management for acute pancreatitis, given his elevated serum lipase and amylase levels (Table 1). His chest pain resolved within 24 hours of admission, accompanied by resolution of his ECG changes with normokalemia. His epigastric pain subsided over the next 24–36 hours. Computed tomography angiography (CTA) of the coronary circulation showed that the dominant R-ACAOS coursed between the aorta and the pulmonary trunk (Fig. 3) and demonstrated no evidence of coronary calcium or atherosclerotic changes. The patient underwent exercise testing with myocardial perfusion imaging prior to hospital discharge. He exercised for 10:04 minutes on a regular Bruce protocol (99% predicted maximal heart rate) with normal BP response and without ECG changes. Myocardial perfusion images showed no evidence of myocardial ischemia.

## Discussion

Concurrent chest and epigastric pain, especially in patients with ECG changes suggestive of myocardial ischemia or injury, can pose a diagnostic challenge. Our patient presented with chest pain and dynamic ECG changes mimicking critical stenosis of the LAD coronary artery, accompanied by epigastric pain, elevated amylase and lipase levels, and hypokalemia. The patient’s chest pain was concerning for unstable angina with dynamic ECG changes consistent with Wellens syndrome. Hence, despite his normal troponin levels measured by point-of-care assay in the ED and confirmed by high-sensitivity troponin assay in the central laboratory (Table 1), as well as no wall motion abnormality by bedside transthoracic echocardiography during chest pain, an invasive approach was taken to rule out a critical stenosis high in the LAD coronary artery. After resolution of his chest pain and normalization of his ECG changes with normokalemia, his initial ECG changes were attributed to his hypokalemia caused by acute alcohol-induced pancreatitis because his serum amylase and lipase levels were elevated.

Acute ECG changes are present in over half of patients with acute pancreatitis and range from ST-segment and T-wave changes to intraventricular conduction abnormalities [4, 5]. The underlying mechanisms of ECG changes in acute pancreatitis remain unclear. However, several mechanisms have been suggested, including electrolyte abnormalities, coronary vasospasm, exacerbation of underlying cardiac disease, cardiobiliary reflex, coagulopathy, and direct effects of proteolytic enzymes on the myocardium [1, 5–8]. The patient had no cardiac disease or coagulopathy, and his pancreatitis was more likely alcohol-related than due to gallstones. Another potential mechanism could be acute stress-induced cardiomyopathy, which has been described previously in patients with acute pancreatitis [9, 10]. However, transthoracic echocardiography during chest pain and at the time of electrocardiographic changes showed normal left ventricular systolic function without regional wall motion abnormalities. The brevity of ECG changes in our patient (resolution in less than 24 hours) may suggest coronary vasospasm as a likely mechanism; however, the ECG changes were not typical of this condition.

Although the discovery of the R-ACAOS was incidental and not contributing to the patient’s clinical presentation, its interarterial course is considered to confer higher risk for sudden cardiac death, especially when intramural, owing to a sharp angle between the aorta and the ectopic coronary artery, an easily collapsible proximal portion, as well as to an ostial configuration that is usually slitlike in shape [11–14]. Though our patient had an R-ACAOS with an interarterial course and chest pain, which could be a premonitory symptom, his ECG findings were not consistent with a dominant RCA territory ischemia/injury. Furthermore, ostial pathology and an intramural course of the proximal R-ACAOS were excluded by coronary CTA (Fig. 3). Finally, exercise stress testing with myocardial perfusion imaging did not elicit objective or subjective signs of myocardial ischemia.

## Conclusions

This case report highlights the persistent diagnostic challenge over the past century when a patient presents with concurrent angina pectoris and epigastric pain associated with ECG features suggestive of acute myocardial ischemia/injury. Clinicians may consider deferring immediate cardiac catheterization and attribute ECG changes to acute pancreatitis if confirmed by normal cardiac enzymes and elevated levels of lipase and amylase. However, when clinical signs and ECG findings are highly suggestive of myocardial ischemia/injury, immediate noninvasive coronary CTA may be the best approach to make an early diagnosis in patients with angina pectoris and normal cardiac enzymes.

## Data Availability

All data applicable to this case report is provided within the case report, including the table and figures.

## Abbreviations

BP: Blood pressure
CTA: Computed tomography angiography
ECG: Electrocardiogram
ED: Emergency department
LAD: Left anterior descending
R-ACAOS: Anomalous origin of the right coronary artery from the opposite sinus
RCA: Right coronary artery
